# Two reactive behaviors of chondrocytes in an IL-1β-induced inflammatory environment revealed by the single-cell RNA sequencing

**DOI:** 10.18632/aging.202857

**Published:** 2021-04-20

**Authors:** Chenghao Gao, Hongxu Pu, Qian Zhou, Tenghui Tao, Hui Liu, Xuying Sun, Ximiao He, Jun Xiao

**Affiliations:** 1 Department of Orthopedic Surgery, Tongji Hospital, Tongji Medical College, Huazhong University of Science and Technology, Wuhan 430030, Hubei, China; 2 Department of Physiology, School of Basic Medicine, Tongji Medical College, Huazhong University of Science and Technology, Wuhan 430030, Hubei, China; 3 Center for Genomics and Proteomics Research, School of Basic Medicine, Tongji Medical College, Huazhong University of Science and Technology, Wuhan 430030, Hubei, China; 4 Hubei Key Laboratory of Drug Target Research and Pharmacodynamic Evaluation, Huazhong University of Science and Technology, Wuhan 430030, China

**Keywords:** chondrocyte, inflammation, osteoarthritis, IL-1beta, single-cell RNA sequencing

## Abstract

Objective: To investigate the heterogeneous responses of *in vitro* expanded chondrocytes, which were cultured in an interleukin (IL)-1β -induced inflammatory environment.

Method: Human articular chondrocytes were expanded, *in vitro*, for 13 days and treated with IL-1β for 0, 24, and 48 h. Cells were collected and subjected to single-cell RNA sequencing. Multiple bioinformatics tools were used to determine the signatures that define chondrocyte physiology.

Results: Two major cell clusters with distinct expression patterns were identified at the initial phase and were with heterogeneous variation that coincides with inflammation progress. They transformed into two terminal cell clusters one of which exhibited OA-phenotype and proinflammatory characteristics through two paths, “response-to-inflammation” and “atypical response-to-inflammation”, respectively. The involved cell clusters exhibited intrinsic relationship with cell types within native cartilage from OA patients. Genes controlling cell transformation to OA-phenotype were relating to the tumor necrosis factor (TNF) signaling pathway via NFKB, up-regulated KRAS signaling and the IL2/STAT5 signaling pathway and pathways relating to apoptosis and reactive oxygen species.

Conclusion: The *in vitro* expanded chondrocytes under IL-1β-induced inflammatory progression behave heterogeneously. One of the initial cell clusters could transform into a proinflammatory subpopulation through a termed response-to-inflammation path, which may serve as the core target to alleviate OA progression.

## INTRODUCTION

Osteoarthritis (OA) is characterized by cartilage degradation, featuring extracellular matrix (ECM) loss, and has been associated with disordered chondrocyte function [1]. The cartilage architecture and ECM composition are regulated by chondrocytes, in response to chemical or physical microenvironment alterations [2]. Accumulating evidence has indicated that inflammation is a critical component of OA disease pathogenesis. Enhanced catabolic reactions and apoptosis in chondrocytes have been observed in inflammatory cartilage, and chondrocytes in inflammatory environments tend to regenerate abnormal ECM architectures, resulting in an imbalance between ECM production and degradation [3–5]. The pharmaceutical inhibition of inflammatory pathways has demonstrated some efficacy for delaying OA progress but has not been able to prevent or restore disrupted cartilage [6, 7]. However, understanding the effects of local and systemic inflammatory environments during OA disease progression and in response to cartilage transplantation [5, 8], particularly the effects of inflammation on chondrocyte pathophysiology, and the metabolic mechanisms that result in OA development are particularly important for developing methods to delay disease progression.

Intensive metabolic studies have focused on OA tissue as a whole [9–11], and traditional bulk cartilage sequencing analyses have identified several genes and pathways that were altered in association with inflammation [12]. However, these studies have revealed limited details regarding the distinctions underlying chondrocyte responses, and whether chondrocyte responses to an inflammatory environment are heterogeneous remains unknown. Furthermore, how the disease is initiated at the cellular level during early stages and how it evolves into dysfunction or degeneration at the organ level has not yet been fully explained. Single-cell analyses can contribute greatly to the fundamental understanding of disease development and progression, which has been demonstrated widely in studies examining tumor heterogeneity [13–15] and embryo development [16, 17]. Single-cell RNA sequencing (scRNA-seq), combined with advanced data science, has been a useful technology for examining the transcriptome, allowing the depiction of the compositions and alterations of different cell clusters within an intricate tissue [18] and revealing cell states that may arise transiently or permanently during the gradual processes of disease development, controlling the dynamic transitions that result in pathologic phenotypes [19, 20]. These methods enable the exploration of critical molecules and pathways associated with different cell identities, during the degenerative progress in an inflammatory environment. Fuchou Tang et al. has constructed an RNA library of 1,464 chondrocytes, isolated from native cartilage at different stages from OA patients and depicted the evolution of each cluster in OA progress [21]. However, the heterogeneity of genetic background and individual variation increased the complexity of the specific mechanism of cell transformation, hindering the exploration of core function switch involved in OA.

To clarify how chondrocytes transform in an inflammatory environment at the single-cell level and to explore the dynamic key pathways and cell components involved in functional changes, our study applied scRNA-seq to chondrocytes cultured in an IL-1β-induced inflammation, at three time points. Bioinformatic analyses were conducted to illustrate the detailed heterogeneous cellular responses, to reveal the inflammation-associated progression pathways through which cells gradually lose their homeostasis and gain the disease-related phenotype, and to identify the critical factors necessary for inflammation progression, which will provide additional information for the design of precision medicine strategies to treat OA.

## RESULTS

### Identification of inflammation-responding cell clusters and their expression signature

**Figure 1:**
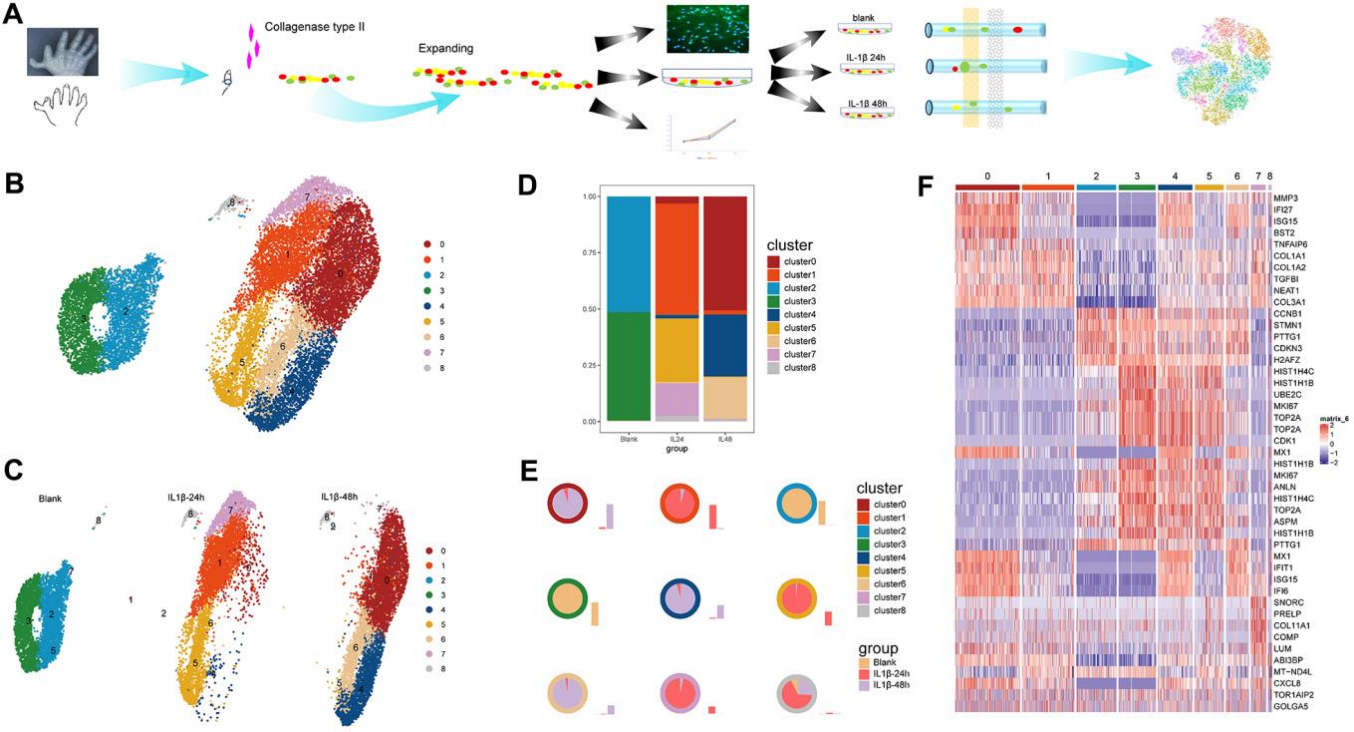
**Work flow and identification of cell clusters.** (**A**) A schematic of the process of species access and data collection. (**B**) The u-map analysis of the integrated data resulted in the identification of nine distinct cell clusters. (**C**) The distributions of the cell clusters on each sample; different colors represent different cell clusters and each plot indicates one cell. (**D**) The stacked chart of the proportions of the distinct cell clusters in the three samples. (**E**) The distribution ratio and quantity in samples of each cell cluster. The colors of the outer circle of each pie chart represent cell clusters. Each pie chart illustrates the ratio of one certain cluster in three samples and the accompanying histogram shows the quantity change of one certain cluster in three samples. (**F**) The graphical heatmap of the top 5 differentially expressed genes for each cell cluster.

The intact cartilage was isolated and digested from discarded finger tissue after excision surgery from the same donor. The *in vitro* expanded chondrocytes were then subjected to designed experiments. Figure 1A shows a schematic showing the process of species access and data collection along with which cell proliferation and morphology were also explored. Enhanced catabolism and induced inflammatory in chondrocyte under IL1β stimulation were confirmed by qPCR which showed up regulated gene expression of MMP1, MMP3, MMP13 and ADAMTS5, and genes of inflammatory factors like TNF and IL6 (Supplementary Figure 1). The well-distributed cytoskeleton was observed, and several cellular pseudopodia could be identified, although they were not quite as distinct, in both articular chondrocytes (ACs) treated with IL-1β and controls (Supplementary Figure 2A). Correspondingly, no significant difference in proliferation activity was identified in ACs treated with IL-1β and control ACs, after a 3-day culture (Supplementary Figure 2B). During a short-term inflammatory stimulation period, ACs demonstrated few changes in proliferation rate or macroscopic structure. Thus, the following analysis would mainly focus on the function transformation of ACs.

After quality control, 4,703, 6,170, and 7,348 cells were recruited in downstream analysis after individually sequenced using scRNA-seq to characterize their transcriptomes. The u-map analysis of the integrated data resulted in the identification of nine distinct cell clusters, with numeric distinction among separate subpopulations (Figure 1B). The separation distance between all the identified cell clusters could easily distinguish the control cells and IL-1β stimulated cells among which the former was mainly consisted of clusters 2 and 3 and the latter of clusters 0, 1, 4, 5, 6,7. This was further confirmed by the split plot of cells from each sample (Figure 1C) which showed that clusters 1, 5 and 7 enriched in the 24-h sample, and clusters 0, 4, and 6 were mainly from the 48-h sample while the cluster 8 consistently existed in all the samples. Here, we defined the cell cluster by different gene expression patterns in order to explore the function transformation from the initial to the terminal phase.

The two dominating clusters in control sample shifted dramatically into the six clusters in stimulated samples with significant increases or decrease in the proportions of certain clusters following the IL-1β induction of inflammation in the culture environment (Figure 1D, 1E). Specifically, the proportions of cells grouped in clusters 1,7, and 5 increased sharply, from baseline at 0-h to highest quantity level in the 24-h sample, whereas the proportions of cells grouped in clusters 2,3 almost disappeared after IL-1 stimulation. Subsequently, clusters 0,4, and 6 gradually took place of the previously dominating clusters 1, 5 and 7 along with the continuing inflammation environment and constituted the main composition in the 48-h sample. So, the clusters 0,4, and 6 may serve as the potential inflammation-special cell cluster in the terminal phase.

There is a continuous but heterogeneous dynamic changes of cell function among these cell clusters along with the IL1β stimulation. Differential gene expression analysis was conducted among the u-map-separated clusters, and the graphical representation (heatmap) indicated the top 5 differentially expressed genes for each subpopulation (Figure 1F). Notably, although the gene expression patterns were distinct among clusters with marked fluctuations in cell quantities, the specifically high-expressed genes among clusters 0, 4 and 6 were most commonly associated with the metabolism of the ECM such as *MMP3*, COL1A1, COL1A2, and *COL3A1* and inflammatory factors like IFI27, TNFAIP6, TGFBI. Interestingly, clusters 1,5 and 7 shared some common high-expressed genes with clusters 0,4, and 6, most of which were relating to collagen synthesis. Although these cell clusters exhibited dynamic changes in terms of cell quantities and genetic functions in different samples, cluster 8 showed a relatively stable status independent of stimulation whether in cell numbers or gene expression pattern.

### The correlation between clusters-special genes and functional gene sets and previously-identified marker genes of subpopulation of chondrocytes

**Figure 2:**
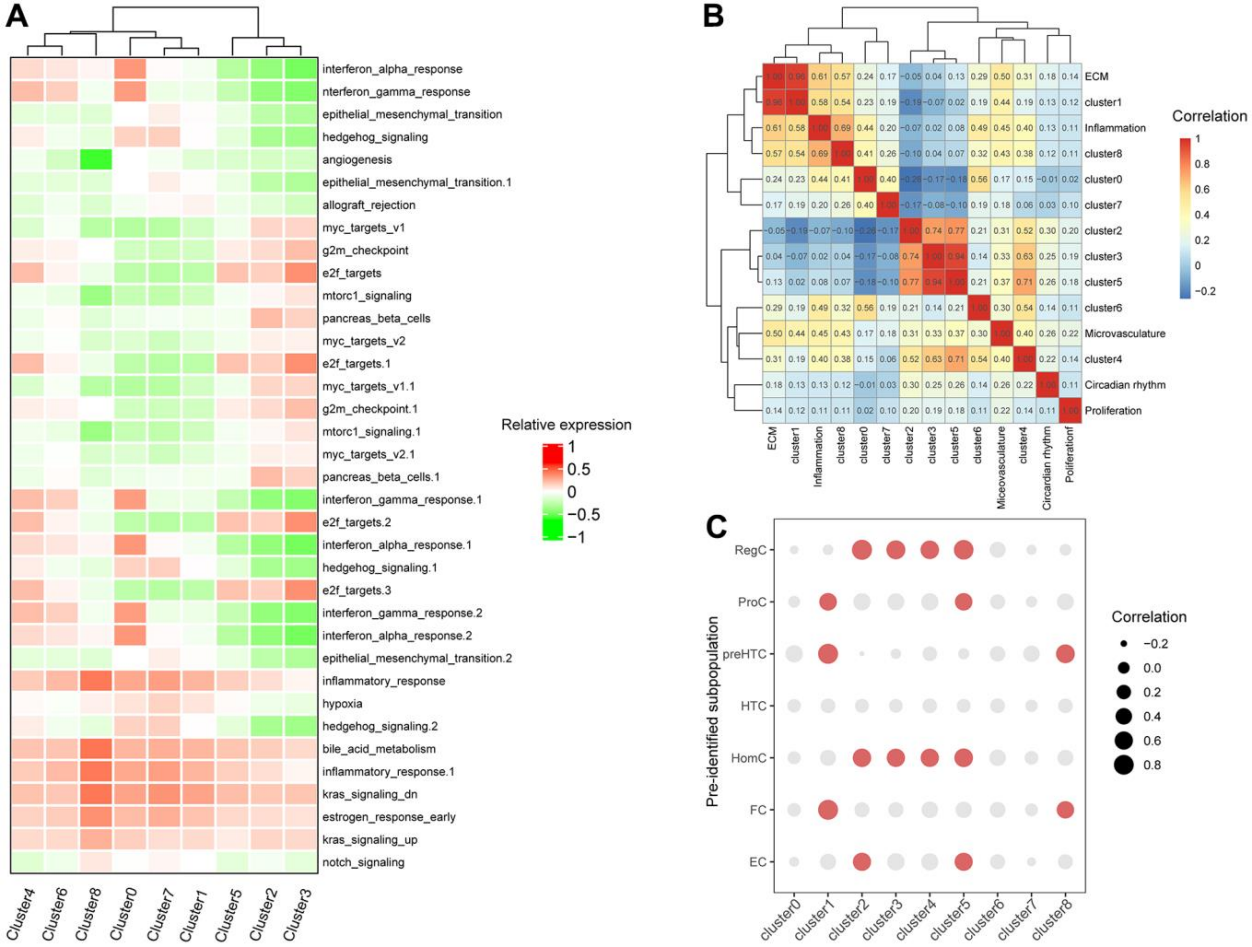
**Correlation between expressions of clusters-special genes and functional gene sets and previously-identified marker genes of subpopulation of chondrocytes.** (**A**) The heatmap of enriched pathways in clusters by GSVA. (**B**) The bubble chart of correlations between clusters-special genes and previously-identified marker genes of subpopulation of chondrocytes. (**C**) The heatmap of correlations between subpopulation of chondrocytes and constructed gene sets.

To classify the function of each cluster, we first referred pathway enrichment based on gene set variation analysis, and then calculated the correlations between the expression signature of the 9 clusters and constructed functional gene sets. Pathway enrichment analysis revealed an increasing relation to inflammatory response from clusters in original state like cluster 2 and 3 to the terminal clusters such as clusters 0 and 4 while clusters 1,7, and 0 closely linked (Figure 2A). The heatmap of correlation between cluster-specific expressed genes and constructed functional gene sets also indicated that cluster 1-specific genes were highly related to the inflammatory gene set among the major cell clusters in stimulated samples. However, gene sets containing the genes that regulate circadian rhythm, proliferation, or microvasculature were not significantly associated with any cluster-special genes (Figure 2B).

The subpopulation of chondrocytes was previously identified by as hypertrophic chondrocytes (HTCs), prehypertrophic chondrocytes (preHTC), fibrocartilage chondrocytes (FC), regulatory chondrocytes (RegC), homeostatic chondrocytes (HomC), effector chondrocytes (ECs), and proliferative chondrocytes (ProCs). Correlation between cluster-specifical genes expression and previously-identified marker genes expression of subpopulation of chondrocytes showed that cluster 1 was associated with preHTC and FC (cor = 0.82), cluster2 was highest related with RegC (cor = 0.83) and cluster 3 with HomC (cor = 0.60) and RegC (cor = 0.68), cluster4 with HomC(cor = 0.60) and RegC (cor = 0.63), cluster5 with RegC (cor = 0.77) and HomC (cor = 0.64), cluster 8 with preHTC (cor = 0.66) (Figure 2C and Supplementary Figure 3). Notably, earlier appeared clusters 2,3 and 5 were all has a similarity with RegC and HomC while the terminal clusters 0 and 7 have no obvious relationship with any predefined subpopulations. The specific correlation heatmap was presented in Supplementary Figure 3.

### The response-to-inflammation paths constructed by single-cell trajectory analysis

**Figure 3:**
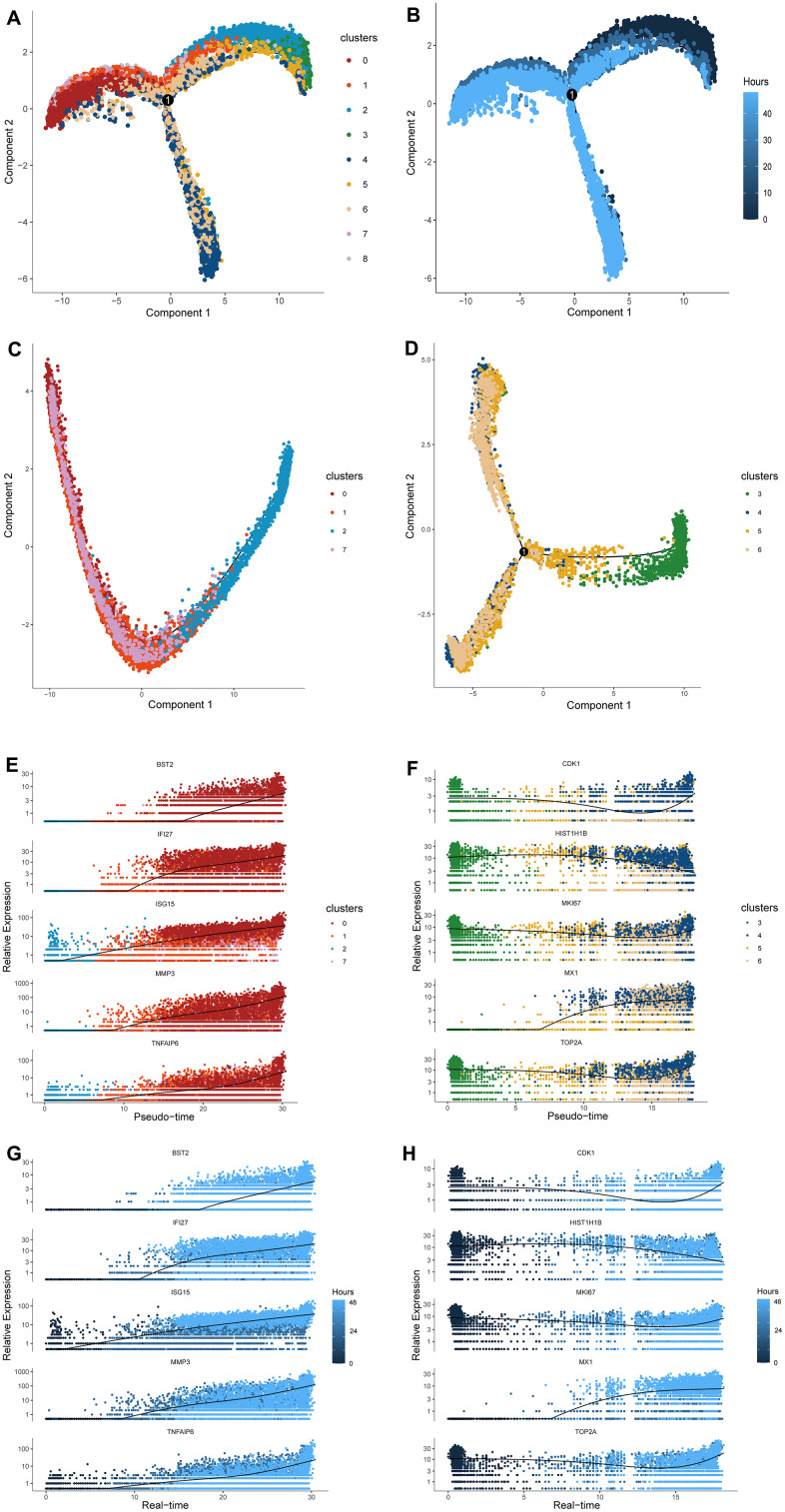
**Cell distribution along pseudo-time trajectory and construction of the response-to-inflammation path.** (**A**) A trajectory with three branches and one connecting points was constructed by Monocle 2. (**B**) The time-line of the pseudo-time trajectory; the darker blue indicates earlier pseudo-time. (**C**) The distribution of cluster2, 1, 7, and 0 along the pseudo-time trajectory. These cells compromised a complete evolutionary route without branches. (**D**) The distribution of cluster3, 5, 6, and 4 along the pseudo-time trajectory. These cells compromised a different evolutionary route from the former with differentiated branches. (**E**–**G**) The expression level of specific representative genes of terminal cluster 0 in cluster2, 1, 7, and 0 are highlighted along the response-to-inflammation path and real-time line. (**F**–**H**) The expression level of specific representative genes of terminal cluster 4 in cluster3, 5, 6, and 4 along the atypical response-to-inflammation path and real-time line.

A trajectory, with three main branches and one connecting points, was constructed by Monocle 2, in which cells were grouped into three states (Figure 3A and Supplementary Figure 4A). The trajectory resulted in two paths along with that cells transformed differently, which was further validated below. We found the cells in clusters 2 and 3 to be distributed in the earlier stage of the trajectory, which covered state 1 and was enriched with most of the cells from control sample, whereas the cells in clusters 1,7, and 0 were located in the later phase and extend into the ending along one branch (Supplementary Figure 4B and Figure 3B). Notably, the upregulated genes in cell clusters 1 and 7 consistently enriched in pathways and functional gene sets correlated with inflammation and ECM as depicted above (Figure 2A, 2B) and were primarily enriched in states 2 corresponding with one of the end directions of the pseudo-time trajectory. Thus, the increasing enrichment of end-state cells, the cluster-0 cells, were considered to be the final inflammation-responded chondrocytes during the late inflammatory stage. Therefore, we termed the path, in which cells were distributed from the root state 1 to the end state 2, the “response-to-inflammation path.”

When examining changes in the transcript abundance of the inflammation genes in each cluster along the peseudotime trajectory, gene expression was observed to fluctuate with IL-1β stimulation but the trend was observed in one path. Specifically, the gradually increasing expression of *BST2*, *IFI27*, *ISG15*, *MMP3*, and *TNFAIP6,* most of which were inflammatory factors, were not only reflected by the order of clusters 2,1, 7, and 0, namely the “response-to-inflammation path”, but also match the time line of stimulation (Figure 3E, 3G). Namely, this peseudotime trajectory was consistent with the actual time line designed in this study.

The other path illustrated the evolving route from root cells of cluster 3 to the other ending cells of clusters 5, 6, and 4, of which the enriched pathways have relatively little relationship with inflammation and ECM metabolism (Figure 2A, 2B). Considering these cells ended at a different state comparing with the above “response-to-inflammation path”, this trajectory was termed as “atypical response-to-inflammation path”. Correspondingly, highly expressed genes of the terminal cluster 4 such as *CDK1*, *HIST1H1B*, *MKI67*, *MX1*, and *TOP2A*, showed a significant variation but not an exact trend along the “atypical response-to-inflammation path” both in pseudo-time and real-time (Figure 3F, 3H).

### The characteristics of response-to-inflammation paths validated by gene function annotation and the identification of the key genes depending cell fate

**Figure 4:**
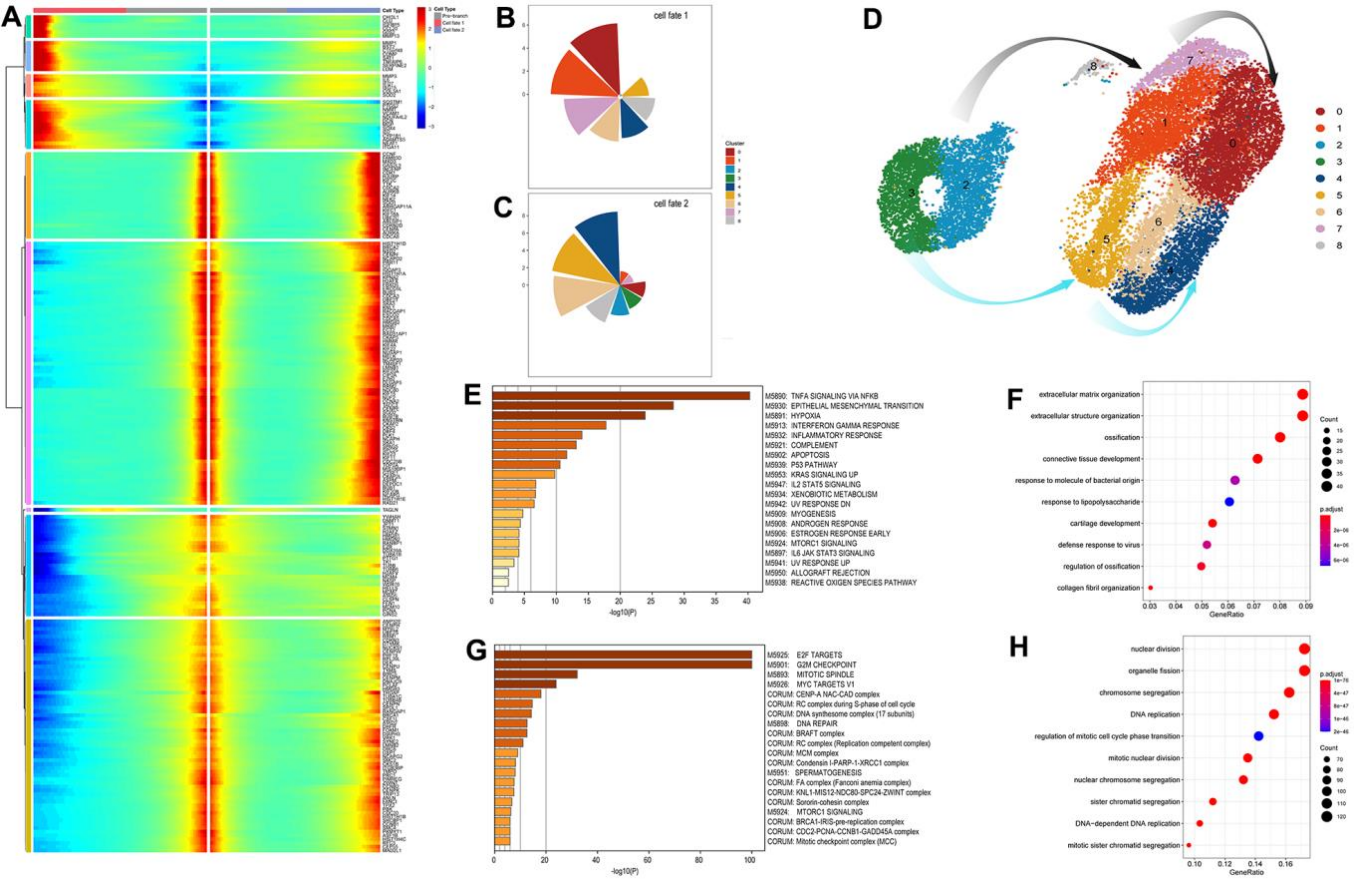
Identification and analysis of genes involve in the connected point in the pseudo-time trajectory (**A**) The expression patterns of the genes associated with cell fate at point 1. Cell fates depend on the up- or down-regulation of these genes. (**B**, **C**) The pie chart of the proportions of the distinct cell clusters in cell fate 1 and 2. (**D**) The exhibition of cell transformation along two defined pathways. The grey arrows indicate the response-to-inflammation path and the azure arrows indicate the atypical response-to-inflammation path. (**E**, **F**) Significant signaling pathways and biological processes identified by Gene set enrichment analysis (GSEA) and gene ontology (GO) analysis conducted on fate 1 (the response-to-inflammation path) associated genes. (**G**, **H**) Significant signaling pathways and biological processes identified by Gene set enrichment analysis (GSEA) and gene ontology (GO) analysis conducted on fate 2 (the atypical response-to-inflammation path) associated genes.

To explore changes in cell function as cells evolve into the final state along this trajectory, we evaluated the gene expression pattern associated with point 1 where cells separately begin moving into different states, one of which mainly contained the inflammation amplification clusters and the other was atypical response-to-inflammation path. The expressed genes associated with cell fate at point 1 were shown in Figure 4A. Specifically, cells with up-regulated genes such as *CHI3L1*, *CLU*, *IGFBP5*, *CCL20*, *G0S2*, *MMP13*, *MMP1*, *BST2*,*C15orf48*, *COMP*, *SAT1*, *TNFAIP6*, *SERPINE2*, *LUM*, and *MMP3* were assigned to cell fate1 which also represented the direction of the response-to-inflammation path while those with up-regulated genes like *CCNF*, *FAM83D*, *HIST1H1D*,*BRCA2*, *YWHAH*, *DNMT1*, *ANP32E*, and *CENPH* were allocated to cell fate2 following the atypical response-to-inflammation path. Consistently, the fate1 cells were mainly constructed by clusters 0, 1, and 7 while the fate2 cells mostly obtained clusters 4, 5, and 6 (Figure 4B, 4C), confirming the existence of the defined two paths through which chondrocytes reacted differently (Figure 4D).

The top pathways enriched in fate1 associated genes showed a corresponding results and are listed in figure 4E–4H. Notably, significantly enriched pathways relating to complement and allograft rejection were suggested that immune system may also play a role in the cartilage degeneration apart from the common inflammation regulatory pathways, such as the tumor necrosis factor (TNF) signaling pathway via NFKB, up-regulated KRAS signaling and the IL2/STAT5 signaling pathway. And pathways relating to apoptosis and reactive oxygen species indicated more specific biological processes involved in the cell’s transformation along the response-to-inflammation path. However, top pathways enriched by fate 2 associated genes mainly participated the cell cycle regulation such as E2F target, G2M checkpoint and mitotic spindle, representing a completely different cell state from cells in fate 1.

A gene ontology (GO) analysis conducted on these fate-depending genes is depicted in Figure 4F, 4G. Specifically, genes regulating cell transformation toward fate1 were more responsive to extracellular modification, with the most significantly enriched genes associated with extracellular structure organization during biological processes, and other classical phenotype in OA chondrocytes such as the ossification, connective tissue development, cartilage development, and collagen fibril organization, all involved in ECM catabolic functions. In comparison, genes regulating cells to the state 3 were more enriched in cell proliferation like nuclear division, organelle fission, chromosome segregation and DNA replication.

## DISCUSSION

The elementary functional unit of a tissue is the genetically and epigenetically variable cell, which manages the network that regulates all events in the biology or pathology of any particular organ tissue [13]. OA comprises a range of mild to advanced cartilage lesions and morphologic changes, combined with a long-lasting inflammatory microenvironment that contributes to cartilage degeneration, especially chondrocyte dysfunction, in the diseased joint [22, 23]. Consequently, we attempted to identify onset cells that are highly sensitive to pathogenic factors during the early stages of the disease. Generating scRNA-seq profiles for each sample in the IL-1β-induced inflammation that is widely used to induce an inflammatory environment when studying the degeneration mechanism of chondrocyte [24] and then evaluating alterations among the cell states facilitated the investigation of underlying progress route, at a high resolution.

Our study indicated the existence of two major cell clusters with distinct expression patterns and heterogeneous response towards IL-1β stimulation, and these cells switches rapidly in both quantity and gene expression patterns over time. Consistent with the findings of Tommy et al., cells cultured *in vitro* showed no apparent distance among clusters as they distributed very close in the blank sample in this study; however, expression heterogeneity exists when looking into the specific gene expression, even though chondrocytes are supposed to dedifferentiated into homogeneous cells [8]. The intrinsic heterogeneity was further revealed when cells were stimulated with IL-1β. With no evidence of varied cell morphology or proliferative abilities, the significant transformation and dramatically different cell status revealed this heterogeneity as these variations in gene expression patterns result in more cell clusters with altered cell function and cell ratio of subpopulations, although no definite markers for cell type were identified. Genetically distinct cells in this study can represent an individual cell’s susceptibility to inflammation because it has got rid of the association with various situations, dimensions, or disease states [25, 26].

The intrinsic heterogeneity enveloped in dedifferentiated chondrocytes may be the persistence of characteristics of the subpopulation of the native cartilage, and would be amplified through some stimulation like IL1-β induced inflammation. Correlation between cell clusters and previously-identified subpopulations indicated close linkage between native chondrocytes with those cultured *in vitro* according to the correlation analysis conducted on the clusters we found and those of Fuchou Tang et al.’s report. Notably, we observed HomCs and RegCs shared some common characteristics with clusters 3 and 4 while cluster 1 contained similar transcriptome features to FCs and preHTCs. The preHTC populations control the typical hypertrophy phenotype chondrocytes (HTCs) of OA cartilage and FCs were mainly enriched in OA cartilage while RegCs and HomCs are characterized by regulating cell functions during OA progression [21]. It was consistent with the intermediate role of these clusters during the progress of chondrocytes under IL1-β stimulation. The top genes identified in the signature expression profile of cluster 0 included *MMP3*, *IFI27*, ISG15, BST2, and *TNFAIP6*, among which, *MMP3* produces enzymes that can degrade all structures of the extracellular matrix, whereas genes, such as *ISG15* and *TNFAIP6* are accepted as inflammatory-related genes [27, 28] and *BST2* [29] and *IFI27* [30] are involved in innate immunity induced by pro-inflammatory cytokines. Other collagen metabolic genes, such as *COL1A1, COL1A2,* and *COL3A1*, also showed high level of expression in cluster 0, further supporting the fact that the terminal cluster 0 stays in inflammatory and ECM-metabolic status. By contrast, the other ending cluster 4 exhibited very different behavior from cluster 1 with up-regulated genes relating to cell cycle rather than inflammation or extracellular matrix. Therefore, the subset of chondrocytes in cluster 0 may represent the major components responsible for the pro-inflammatory process and ECM degradation. Additionally, genes that regulate circadian rhythms and microvasculature are also thought to play important roles during OA progression [31–33] However, no evidence was identified to support the involvement of circadian- or microvascular associated genes, in any special cluster, during the response to inflammation. The excessively long *in vitro* culture time (13 days) and the dedifferentiation culture process may have some impact on the cell function of circadian rhythm. Although there was no proliferation-special cluster identified, the up-regulated genes relating to cell cycle indicted the involvement of cell proliferation and a more recent study found that CD44, JUN and FN1 were not only regulating proliferation but also indicating OA progress by reanalysis the published single cell sequencing data [34].

The pseudo-time analysis in Monocle2 illustrated two paths of the transcriptional alterations that occur in cells under the IL-1β-induced inflammation, indicting two continuous and heterogeneous processes, one of which is highly associated with the initiation and advancement of cartilage degeneration. These results are consistent with the complexity and heterogeneity of disease progression in OA chondrocytes [35]. Furthermore, the generated response-to-inflammation path, along which cells were distributed from the early root to the inflammatory end, may represent the progression of chondrocytes, transforming into cell clusters that promote inflammation and therefore, induce abnormal ECM. Because point 1 represents a pivotal junction where cells transform into the two distinct end states, we hypothesized that the highly variable genes identified during this fate-determined step may be involved in controlling inflammatory progression. Some molecules and signaling pathways revealed by this research have been explored by recent studies, such as the ROS/oxidative stress signaling [36], apoptosis [37] and the activation of nuclear factor (NF)-κB signaling and TNF signaling pathways, all involved in the regulation of inflammation and cell death. The immune role was also suggested to contribute to osteoarthritis [38]. Together with the gradual increase in expression levels observed for well-known pro-inflammatory genes from the original clusters to cluster 0, the response-to-inflammation pathway appears to reflect transcription variations that underlie inflammatory progression. This finding is partly consistent with the findings of a more recent study [39] in which Grandi et al. identified two rare inflammation-modulating subpopulations, one of which was defined as an inflammation-amplifying population, characterized by the increased expression of IL-1 receptor 1 and TNF receptor II. Although the specific inflammatory molecules between these two studies differ, our study can be viewed as complementary evidence regarding an inflammation-specific subpopulation of chondrocytes when cultured *in vitro* and revealing the transcript signatures associated with changes in chondrocyte physiology. Therefore, functional properties of chondrocytes, both *in vitro* and *in* *vivo* could provide information at the single-cell level for the further development of putative drugs aimed at the key molecules expressed in the inflammation-specific subpopulation.

In summary, the present study provides a high-dimensional transcript landscape for *in vitro* expanded chondrocytes cultured in an IL-1β-induced inflammatory environment, revealing multiple subpopulations, with diverse functions. Collectively, the analysis results demonstrated the sophisticated alterations that occur in original cell clusters during inflammatory progression and the existence of a response-to-inflammation path with a terminal inflammation-specific subpopulation of chondrocytes, suggesting that this subpopulation, together with the genes that determine this subpopulation, could represent novel therapeutic targets for OA. However, additional single-cell data from a larger cohort of patients with OA and the further exploration of molecular events, such as single-cell proteomics, remain necessary to refine panels that accurately define the inflammatory path and specific subpopulation, and to establish reliable precision medicine strategies to treat OA.

## MATERIALS AND METHODS

### Ethics statement

Investigation has been conducted in accordance with the ethical standards and according to the Declaration of Helsinki and according to national and international guidelines and has been approved by the Committee for Medical Research Ethics of Tongji Hospital.

### Isolation and culture of human AC

ACs, obtained from an interphalangeal joint of a polydactyl child, were isolated from discarded finger tissue after excision surgery. All cells used during single-cell sequencing were obtained from the same donor. The isolated cartilage was intact, containing all cartilage zones, and was used within 6 h of dissection. Briefly, the cartilage was shredded into small pieces, digested with collagenase type II (Sigma-Aldrich, St. Louis, MO, USA) for no more than 4 h, and seeded in culture medium consisting of DMEM/F12 (Thermo Fisher Scientific/ Gibco, Waltham, MA, USA) and containing 10% human platelet lysate-rich plasma (hPLP), 100 units/mL penicillin, and 100 μg/mL streptomycin (Sigma-Aldrich). ACs were cultured for 13 days (three passages). The obtained cells were reseeded in six-well plates and treated with human IL-1β (h-IL-1β) (10 μg/mL) for 0, 24, and 48 h. Informed consent has been obtained. All experiments were conducted under aseptic conditions.

### Cell proliferation and morphology

Cell counting kit 8 (CCK8, Boster Biological Technology Co., CA, USA) was used to test the proliferative activity of ACs cocultured with h-IL-1β (10 μg/mL) for 24, 48, and 72 h. Approximately 2 × 10^4^ ACs, in 1 mL DMEM/F12, were seeded in a 24-well plate and cultured at 37° C, in a 5% CO_2_ environment, for 24, 48, and 72 h. The culture medium was refreshed every other day. The absorbance of the final reaction solution, which was prepared according to the manufacturer’s instructions, was measured using a spectrophotometer (Bio Tek Instruments Inc., VT, USA).

Phalloidin (Sigma-Aldrich, USA) and 4′,6-diamidino-2-phenylindole (DAPI), Boster Biological Technology Co., CA, USA) were used to the visualize cell skeleton and cell nuclei, respectively. Briefly, ACs in 24-well plates treated with h-IL-1β (10 μg/mL) for 0, 24, and 48 h rinsed with phosphate-buffered saline (PBS), and fixed with 4% paraformaldehyde. Then, they were consecutively stained with phalloidin for 30 min and DAPI for 15 min in the dark. A fluorescence microscope (Nikon Ti-S, Tokyo, Japan) was used to observe changes in cell morphology, after air drying at room temperature.

### The catabolism-related and inflammatory gene expression

Four critical catabolism-related genes, MMP1, MMP3, MMP13 and ADAMTS5, and two inflammatory genes, IL6 and TNF, were examined. The quantitative reverse transcription PCR (qRT-PCR) were applied to measure the gene expression levels. Briefly, cells were cultured in 12-well plates treated with h-IL-1β (10 μg/mL) for 0, 12, 24, and 48 h. The total cell biomass from three randomly selected samples in each timepoints were lysed with Trizol (Invitrogen, Thermo Fisher, MA, USA), and the Superscript II first-strand cDNA synthesis kit (Takara, Beijing, China) were used to reverse transcribe RNA. The SYBR Green (TOYOBO Biotech Co., Osaka, Japan) kit was used to prepare PCR mix and a Bio-radMyiQ2 thermocycler (Bio-Rad Laboratories Inc., Singapore) was used for quantification. All the primers that were used are listed in Table 1.

**Table 1:** The primers used for PCR of catabolism-related and inflammatory genes.

**Gene**	**Forward primer (5′-3′)**	**Reverse primer (5′-3′)**
MMP1	AAAATTACACGCCAGATTTGCC	GGTGTGACATTACTCCAGAGTTG
MMP3	AGTCTTCCAATCCTACTGTTGCT	TCCCCGTCACCTCCAATCC
MMP13	ACTGAGAGGCTCCGAGAAATG	GAACCCCGCATCTTGGCTT
ADAMTS5	GAACATCGACCAACTCTACTCCG	CAATGCCCACCGAACCATCT
IL6	ACTCACCTCTTCAGAACGAATTG	CCATCTTTGGAAGGTTCAGGTTG
TNF	CCTCTCTCTAATCAGCCCTCTG	GAGGACCTGGGAGTAGATGAG
GAPDH	GGAGCGAGATCCCTCCAAAAT	GGCTGTTGTCATACTTCTCATGG

### Cell collection and 10x genomic sequencing library construction

The obtained cells reseeded in six-well plates and treated with h-IL1β (10 μg/mL) for 0, 24, and 48 h were harvested at each timepoint. The collected cells were centrifuged at a speed not exceeding 300 × *g* and then washed three times in PBS. The cell precipitation was resuspended by 1 mL 1x PBS containing 0.04% bovine serum albumin and filtered through a 40-μm cell strainer. After washing twice, 100 μL PBS was added to the cell precipitate, to obtain a single-cell dispersion suspension containing a sufficient number of cells. Cells treated with the Single-Cell 3′ Reagent Kit User Guide v2 (10x Genomics) were then transferred to a Chromium Controller instrument (10x Genomics) to produce single-cell gel bead-in-emulsions (GEMs). After reverse transcription, the GEMs were harvested, and the cDNAs were amplified and cleaned with the SPRIselect Reagent Kit (Beckman Coulter). Indexed sequencing libraries were constructed using the Chromium Single-Cell 3′ Library Kit (10x Genomics), for enzymatic fragmentation, end repair, A-tailing, adaptor ligation, ligation cleanup, sample index PCR, and PCR cleanup. The barcoded sequencing libraries were quantified by quantitative PCR, using the KAPA Library Quantification Kit (KAPA Biosystems). Sequencing libraries were loaded onto a NextSeq500 (Illumina), with a custom sequencing setting (26 bp for read 1 and 98 bp for read 2), to obtain a sequencing depth of approximately 80,000 reads per cell. Sequencing data were aligned to the human reference genome (GRCh38) and processed using the CellRanger 2.1.0 pipeline (10x Genomics).

### Single-cell RNA-seq data preprocessing and clustering of chondrocyte cells

Raw gene expression matrices of all sample (including Blank sample, IL24 sample and IL48 sample) were combined in R (version 4.0.2) and converted to a Seurat object using the Seurat R package (version 3.2.0) [40]. Subsequently, all cells were removed that had either fewer than 500 UMIs, over 25000 or below 200 expressed genes, over 5% UMIs derived from mitochondrial genome, or log10 UMIs of per Gene lower than 0.8. Then we first used the NormalizeData function to normalize library size of each cell with default parameters and the FindVariableFeatures function to select the 3000 most variably expressed genes of each sample [41]. Next, we used the functions FindIntegration Anchors and IntegrateData as recommended by Seurat guidelines to integrated cells from three samples. This procedure created an integrated normalized dataset. We then scaled the integrated dataset using the ScaleData function. PCA analysis (Principal components analysis) was performed and the first 10 PCs, the number of principal components estimated by an Elbow plot, were used further to generated t-SNE dimensionality reductions of RunUMAP function. The clustering process was also performed based on these 10 PCs while graph-based clustering was run using FindNeighbors and FindClusters with a resolution of 0.5. [40, 42]. The corresponding marker genes were defined by FindAllMarkers function of Seurat package.

### The correlation between clusters-special genes and functional gene sets and previously-identified marker genes of subpopulation of chondrocytes

Firstly, we select known gene sets that previous reported, for example, circadian rhythm (*CRY1*, *CRY2*, *CLOCK*, *PER1*, *PER2*, *PER3*, *NR1D1*, and *BMAL1*) [8], Microvasculature (*ARAP3, ADCY4*, *ESAM*, *ERG*, *SLC43A3*, *SOX7*, *PTPRB*, etc.) [43], ECM (*MMP9*, *COL3A1*, *COL1A2*, *COL1A1*, *SOX5*, *PRG4*, etc.), Proliferation (*BAG1*, *ESR1*, *FOXA1*, *GPR160*, *NAT1*, *MAPT*, *MLPH*, and *PGR*), and Inflammation (*IFNG*, *IL5*, *IL6*, *JUN*, *NFKB1*, *STAT1*, *TGFB1*, *TNF*, etc.) [44]. Detailed functional gene programs are listed in Supplementary Table 1. We calculated the average expression of each gene set in the total expression matrix. Then, the expression of corresponding top10 marker genes of each cluster defined by FindAllMarkers function of Seurat packages were also calculated. Correlations between the expression level of known gene sets and the expression level of the top 10 cluster-specific expressed genes were estimated by Pearson’s correlation coefficient. The markers of previously-identified chondrocyte subpopulations offered by previous study were also used to measure the similarity with the cell clusters by the same method The relevant code is shown in Supplementary File 1.

### Single-cell trajectory reconstruction and analysis

MONOCLE (version 2.6.4) was applied to construct single-cell pseudo-time trajectories [45]. Briefly, a set of ordering genes, identified as being differentially expressed between clusters by Seurat, were selected and then passed to Monocle’s reversed graph embedding tool, which performed machine learning to learn a parsimonious principal graph. Dimensional reduction and cell ordering were performed using the DDRTree method and orderCells function with default parameters. The integrated high-dimensional expression profiles were reduced to a low-dimensional space, onto which each cell was projected and ordered in a trajectory. As branch points on the trajectory, the cells were divided into different “states,” and branch-dependent genes were then investigated by GO analysis using Metascape (https://metascape.org/) and Go database and Gene set variation analysis in the GSVA package (version 1.36.2).

### Data available

The row data of single-cell RNA-seq are available at the NCBI’s Gene Expression Omnibus (GEO) data repository with the SRA accession ID PRJNA642343.

## Supplementary Material

Supplementary Figures

Supplementary Table 1

Supplementary File 1

## References

[1] Glyn-Jones S, Palmer AJ, Agricola R, Price AJ, Vincent TL, Weinans H, Carr AJ. Osteoarthritis. Lancet. 2015; 386:376–87. 10.1016/S0140-6736(14)60802-3 25748615

[2] Buckwalter JA, Mankin HJ, Grodzinsky AJ. Articular cartilage and osteoarthritis. Instr Course Lect. 2005; 54:465–80. 15952258

[3] Liu-Bryan R. Inflammation and intracellular metabolism: new targets in OA. Osteoarthritis Cartilage. 2015; 23:1835–42. 10.1016/j.joca.2014.12.016 26521729 PMC4668929

[4] Greene MA, Loeser RF. Aging-related inflammation in osteoarthritis. Osteoarthritis Cartilage. 2015; 23:1966–71. 10.1016/j.joca.2015.01.008 26521742 PMC4630808

[5] Vilá S. Inflammation in osteoarthritis. P R Health Sci J. 2017; 36:123–29. 28915300

[6] Madzuki IN, Lau SF, Abdullah R, Mohd Ishak NI, Mohamed S. Vernonia amygdalina inhibited osteoarthritis development by anti-inflammatory and anticollagenase pathways in cartilage explant and osteoarthritis-induced rat model. Phytother Res. 2019; 33:1784–93. 10.1002/ptr.6366 31033070

[7] Chen J, Gu YT, Xie JJ, Wu CC, Xuan J, Guo WJ, Yan YZ, Chen L, Wu YS, Zhang XL, Xiao J, Wang XY. Gastrodin reduces IL-1β-induced apoptosis, inflammation, and matrix catabolism in osteoarthritis chondrocytes and attenuates rat cartilage degeneration *in vivo*. Biomed Pharmacother. 2018; 97:642–51. 10.1016/j.biopha.2017.10.067 29101808

[8] Karlsen TA, Sundaram AY, Brinchmann JE. Single-cell RNA sequencing of *in vitro* expanded chondrocytes: MSC-like cells with no evidence of distinct subsets. Cartilage. 2019. [Epub ahead of print]. 10.1177/1947603519847746 31072202 PMC8804791

[9] Shet K, Siddiqui SM, Yoshihara H, Kurhanewicz J, Ries M, Li X. High-resolution magic angle spinning NMR spectroscopy of human osteoarthritic cartilage. NMR Biomed. 2012; 25:538–44. 10.1002/nbm.1769 21850648 PMC3299852

[10] Showiheen SA, Sun AR, Wu X, Crawford R, Xiao Y, Wellard RM, Prasadam I. Application of metabolomics to osteoarthritis: from basic science to the clinical approach. Curr Rheumatol Rep. 2019; 21:26. 10.1007/s11926-019-0827-8 31062102

[11] Trachana V, Mourmoura E, Papathanasiou I, Tsezou A. Understanding the role of chondrocytes in osteoarthritis: utilizing proteomics. Expert Rev Proteomics. 2019; 16:201–13. 10.1080/14789450.2019.1571918 30654662

[12] Soul J, Dunn SL, Anand S, Serracino-Inglott F, Schwartz JM, Boot-Handford RP, Hardingham TE. Stratification of knee osteoarthritis: two major patient subgroups identified by genome-wide expression analysis of articular cartilage. Ann Rheum Dis. 2018; 77:423. 10.1136/annrheumdis-2017-212603 29273645 PMC5867416

[13] Baslan T, Hicks J. Unravelling biology and shifting paradigms in cancer with single-cell sequencing. Nat Rev Cancer. 2017; 17:557–69. 10.1038/nrc.2017.58 28835719

[14] Lee HO, Park WY. Single-cell RNA-seq unveils tumor microenvironment. BMB Rep. 2017; 50:283–84. 10.5483/bmbrep.2017.50.6.086 28539161 PMC5498138

[15] Suvà ML, Tirosh I. Single-cell RNA sequencing in cancer: lessons learned and emerging challenges. Mol Cell. 2019; 75:7–12. 10.1016/j.molcel.2019.05.003 31299208

[16] Vento-Tormo R, Efremova M, Botting RA, Turco MY, Vento-Tormo M, Meyer KB, Park JE, Stephenson E, Polański K, Goncalves A, Gardner L, Holmqvist S, Henriksson J, et al. Single-cell reconstruction of the early maternal-fetal interface in humans. Nature. 2018; 563:347–53. 10.1038/s41586-018-0698-6 30429548 PMC7612850

[17] Petropoulos S, Panula SP, Schell JP, Lanner F. Single-cell RNA sequencing: revealing human pre-implantation development, pluripotency and germline development. J Intern Med. 2016; 280:252–64. 10.1111/joim.12493 27046137

[18] Picelli S. Single-cell RNA-sequencing: the future of genome biology is now. RNA Biol. 2017; 14:637–50. 10.1080/15476286.2016.1201618 27442339 PMC5449089

[19] Wagner A, Regev A, Yosef N. Revealing the vectors of cellular identity with single-cell genomics. Nat Biotechnol. 2016; 34:1145–60. 10.1038/nbt.3711 27824854 PMC5465644

[20] Chen X, Teichmann SA, Meyer KB. From Tissues to Cell Types and Back: Single-Cell Gene Expression Analysis of Tissue Architecture. In: Altman RB, Levitt M, eds. Annual Review Of Biomedical Data Science, Vol 1. Palo Alto: Annual Reviews, pp. 29–51, 2018. 10.1146/annurev-biodatasci-080917-013452

[21] Ji Q, Zheng Y, Zhang G, Hu Y, Fan X, Hou Y, Wen L, Li L, Xu Y, Wang Y, Tang F. Single-cell RNA-seq analysis reveals the progression of human osteoarthritis. Ann Rheum Dis. 2019; 78:100–10. 10.1136/annrheumdis-2017-212863 30026257 PMC6317448

[22] Pauli C, Whiteside R, Heras FL, Nesic D, Koziol J, Grogan SP, Matyas J, Pritzker KP, D’Lima DD, Lotz MK. Comparison of cartilage histopathology assessment systems on human knee joints at all stages of osteoarthritis development. Osteoarthritis Cartilage. 2012; 20:476–85. 10.1016/j.joca.2011.12.018 22353747 PMC3348372

[23] Jones KJ, Sheppard WL, Arshi A, Hinckel BB, Sherman SL. Articular cartilage lesion characteristic reporting is highly variable in clinical outcomes studies of the knee. Cartilage. 2019; 10:299–304. 10.1177/1947603518756464 29405742 PMC6585291

[24] Jenei-Lanzl Z, Meurer A, Zaucke F. Interleukin-1β signaling in osteoarthritis - chondrocytes in focus. Cell Signal. 2019; 53:212–23. 10.1016/j.cellsig.2018.10.005 30312659

[25] Shalek AK, Benson M. Single-cell analyses to tailor treatments. Sci Transl Med. 2017; 9:eaan4730. 10.1126/scitranslmed.aan4730 28931656 PMC5645080

[26] Krieg C, Nowicka M, Guglietta S, Schindler S, Hartmann FJ, Weber LM, Dummer R, Robinson MD, Levesque MP, Becher B. High-dimensional single-cell analysis predicts response to anti-PD-1 immunotherapy. Nat Med. 2018; 24:144–53. 10.1038/nm.4466 29309059

[27] Hu Y, Hong XY, Yang XF, Ma RH, Wang X, Zhang JF, Feng Q, Li XG, Sun DS, Li X, Wan HL, Li T, Wang Q, et al. Inflammation-dependent ISG15 upregulation mediates MIA-induced dendrite damages and depression by disrupting NEDD4/Rap2A signaling. Biochim Biophys Acta Mol Basis Dis. 2019; 1865:1477–89. 10.1016/j.bbadis.2019.02.020 30826466

[28] Day AJ, Milner CM. TSG-6: a multifunctional protein with anti-inflammatory and tissue-protective properties. Matrix Biol. 2019; 78:60–83. 10.1016/j.matbio.2018.01.011 29362135

[29] Yi E, Oh J, Kang HR, Song MJ, Park SH. BST2 inhibits infection of influenza A virus by promoting apoptosis of infected cells. Biochem Biophys Res Commun. 2019; 509:414–20. 10.1016/j.bbrc.2018.12.110 30594400

[30] Cheriyath V, Leaman DW, Borden EC. Emerging roles of FAM14 family members (G1P3/ISG 6-16 and ISG12/IFI27) in innate immunity and cancer. J Interferon Cytokine Res. 2011; 31:173–81. 10.1089/jir.2010.0105 20939681 PMC6468951

[31] Jahanban-Esfahlan R, Mehrzadi S, Reiter RJ, Seidi K, Majidinia M, Baghi HB, Khatami N, Yousefi B, Sadeghpour A. Melatonin in regulation of inflammatory pathways in rheumatoid arthritis and osteoarthritis: involvement of circadian clock genes. Br J Pharmacol. 2018; 175:3230–38. 10.1111/bph.13898 28585236 PMC6057898

[32] Mapp PI, Walsh DA. Mechanisms and targets of angiogenesis and nerve growth in osteoarthritis. Nat Rev Rheumatol. 2012; 8:390–98. 10.1038/nrrheum.2012.80 22641138

[33] Korchi AM, Cengarle-Samak A, Okuno Y, Martel-Pelletier J, Pelletier JP, Boesen M, Doyon J, Bodson-Clermont P, Lussier B, Héon H, Sapoval M, Bureau NJ, Soulez G. Inflammation and hypervascularization in a large animal model of knee osteoarthritis: imaging with pathohistologic correlation. J Vasc Interv Radiol. 2019; 30:1116–27. 10.1016/j.jvir.2018.09.031 30935868

[34] Zhang X, Huang N, Huang R, Wang L, Ke Q, Cai L, Wu S. Single-cell rna seq analysis identifies the biomarkers and differentiation of chondrocyte in human osteoarthritis. Am J Transl Res. 2020; 12:7326–39. 10.21203/rs.3.rs-25051/v1 33312370 PMC7724342

[35] Aigner T, Söder S, Gebhard PM, McAlinden A, Haag J. Mechanisms of disease: role of chondrocytes in the pathogenesis of osteoarthritis—structure, chaos and senescence. Nat Clin Pract Rheumatol. 2007; 3:391–99. 10.1038/ncprheum0534 17599073

[36] Lepetsos P, Papavassiliou AG. ROS/oxidative stress signaling in osteoarthritis. Biochim Biophys Acta. 2016; 1862:576–91. 10.1016/j.bbadis.2016.01.003 26769361

[37] Hwang HS, Kim HA. Chondrocyte apoptosis in the pathogenesis of osteoarthritis. Int J Mol Sci. 2015; 16:26035–54. 10.3390/ijms161125943 26528972 PMC4661802

[38] Lopes EB, Filiberti A, Husain SA, Humphrey MB. Immune contributions to osteoarthritis. Curr Osteoporos Rep. 2017; 15:593–600. 10.1007/s11914-017-0411-y 29098574

[39] Grandi FC, Baskar R, Smeriglio P, Murkherjee S, Indelli PF, Amanatullah DF, Goodman S, Chu C, Bendall S, Bhutani N. Single-cell mass cytometry reveals cross-talk between inflammation-dampening and inflammation-amplifying cells in osteoarthritic cartilage. Sci Adv. 2020; 6:eaay5352. 10.1126/sciadv.aay5352 32201724 PMC7069698

[40] Stuart T, Butler A, Hoffman P, Hafemeister C, Papalexi E, Mauck WM 3rd, Hao Y, Stoeckius M, Smibert P, Satija R. Comprehensive integration of single-cell data. Cell. 2019; 177:1888–902.e21. 10.1016/j.cell.2019.05.031 31178118 PMC6687398

[41] Liu B, Li C, Li Z, Wang D, Ren X, Zhang Z. An entropy-based metric for assessing the purity of single cell populations. Nat Commun. 2020; 11:3155. 10.1038/s41467-020-16904-3 32572028 PMC7308400

[42] Macosko EZ, Basu A, Satija R, Nemesh J, Shekhar K, Goldman M, Tirosh I, Bialas AR, Kamitaki N, Martersteck EM, Trombetta JJ, Weitz DA, Sanes JR, et al. Highly parallel genome-wide expression profiling of individual cells using nanoliter droplets. Cell. 2015; 161:1202–14. 10.1016/j.cell.2015.05.002 26000488 PMC4481139

[43] Wallgard E, Larsson E, He L, Hellström M, Armulik A, Nisancioglu MH, Genove G, Lindahl P, Betsholtz C. Identification of a core set of 58 gene transcripts with broad and specific expression in the microvasculature. Arterioscler Thromb Vasc Biol. 2008; 28:1469–76. 10.1161/ATVBAHA.108.165738 18483404

[44] Smillie CS, Biton M, Ordovas-Montanes J, Sullivan KM, Burgin G, Graham DB, Herbst RH, Rogel N, Slyper M, Waldman J, Sud M, Andrews E, Velonias G, et al. Intra- and inter-cellular rewiring of the human colon during ulcerative colitis. Cell. 2019; 178:714–30.e22. 10.1016/j.cell.2019.06.029 31348891 PMC6662628

[45] Qiu X, Mao Q, Tang Y, Wang L, Chawla R, Pliner HA, Trapnell C. Reversed graph embedding resolves complex single-cell trajectories. Nat Methods. 2017; 14:979–82. 10.1038/nmeth.4402 28825705 PMC5764547

