# Supplementary material for: Two reactive behaviors of chondrocytes in an IL-1β-induced inflammatory environment revealed by the single-cell RNA sequencing
Source: Aging (Albany NY). 2021 Apr 20;13(8):11646–64. doi: 10.18632/aging.202857 (PMC8109072; doi:10.18632/aging.202857)
Supplement: Supplementary Figures [file aging-13-202857-s001.pdf]

SUPPLEMENTARY FIGURES

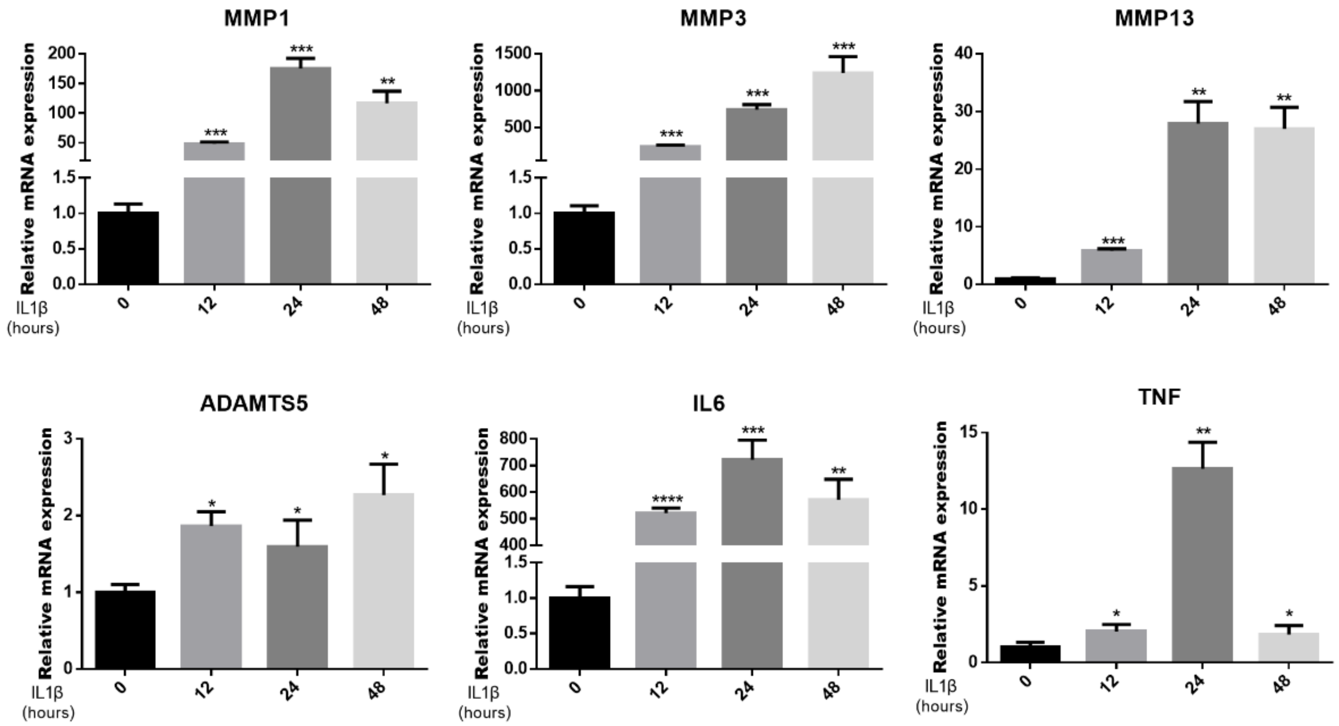

Supplementary Figure 1. The catabolism-related and inflammatory gene expression. Relative expressions of MMP1, MMP3, MMP13, ADAMTS5, TNF and IL6 by chondrocytes cultured under IL1β stimulation for 0-h, 12-h, 24-h and 48-h with all values normalized to expression levels of GAPDH. The expression level of the above genes in groups under IL1β stimulation for 12-h, 24-h and 48-h significantly increased compared with the group under IL1β stimulation for 0-h (the control). *p <0.05; **p < 0.01; ***p < 0.001.

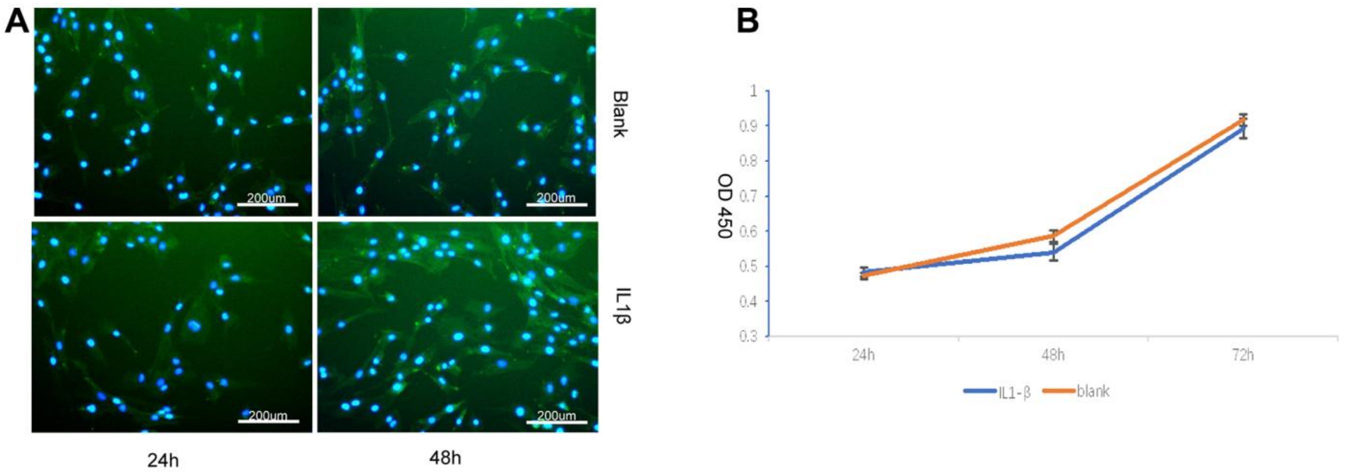

Supplementary Figure 2. Cell proliferation and cell morphology. (**A**) Cell morphology after 24-h and 48-h incubation with or without IL-1β (stained by phalloidin and DAPI). (**B**) Cell proliferation activity tested by CCK8. At 24-h, 48-h, and 72-h, the proliferation activities of cells incubated with or without IL-1β show no significant difference.

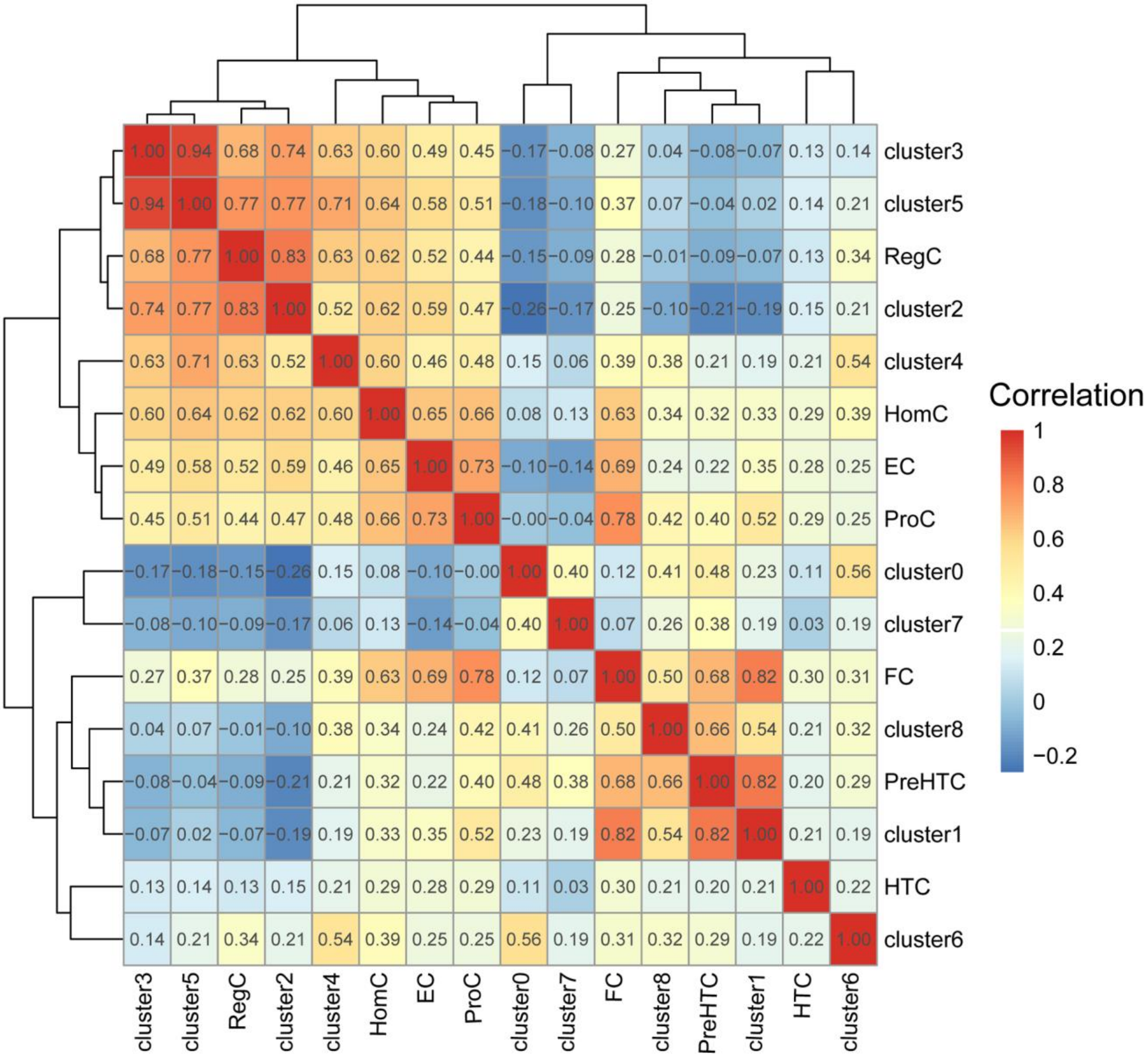

Supplementary Figure 3. The heatmap of correlations between cell clusters and previously-identified cell subpopulations.

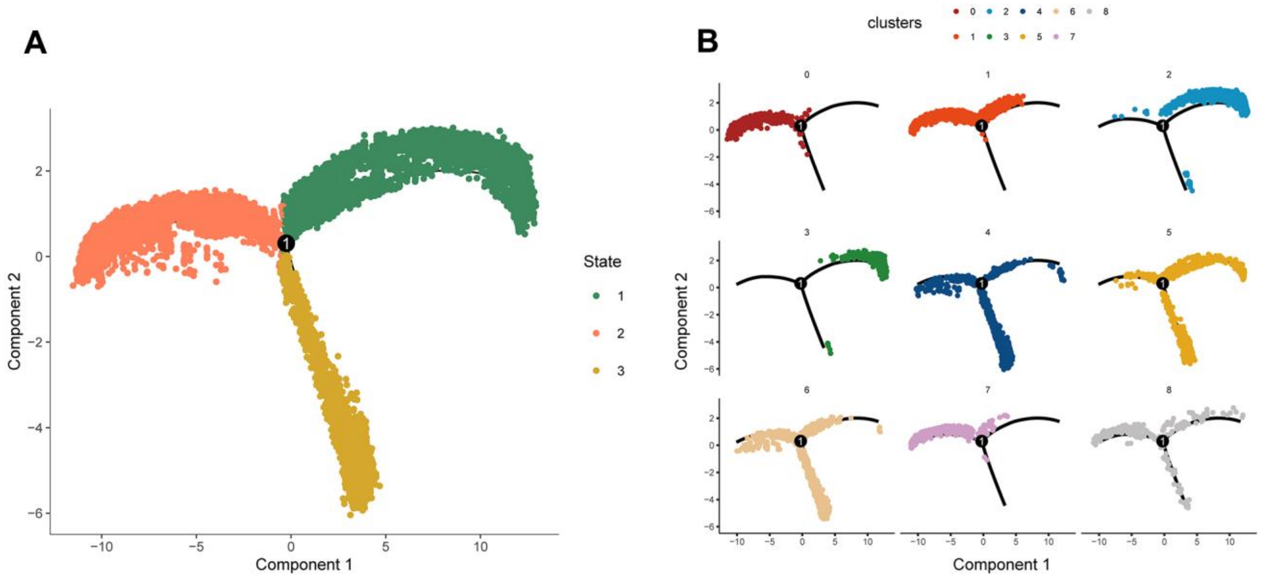

Supplementary Figure 4. The distribution of cells along the pseudo-time trajectory. (**A**) The distribution of each states along the pseudo-time trajectory. (**B**) The split distribution of each clusters along the pseudo-time trajectory.
