# Supplementary material for: Two reactive behaviors of chondrocytes in an IL-1β-induced inflammatory environment revealed by the single-cell RNA sequencing
Source: Aging (Albany NY). 2021 Apr 20;13(8):11646–64. doi: 10.18632/aging.202857 (PMC8109072; doi:10.18632/aging.202857)
Supplement: Supplementary Table 1 [file aging-13-202857-s002.pdf]

SUPPLEMENTARY TABLE

Supplementary Table 1. Genes used to construct functional gene sets.

Function	Gene sets
Circadian rhythm	BMAL1, CLOCK, CRY1, CRY2, NR1D1, PER1, PER2, PER3
ECM	ADAMTS5, B2M, COL11A1, COL1A1, COL1A2, COL3A1, COL5A1, COL5A2, COL6A1, COL6A2, COL6A3, DCN, HAPLN1, ITGA5, ITGB1, ITGB3, MMP13, MMP9, PRG4, SDC4, SOX5
Proliferation	BAG1, ESR1, FOXA1, GPR160, MAPT, MLPH, NAT1, PGR
Microvasculature	ACKR2, ACVRL1, ADCY4, AFAP1L1, ARAP3, C1orf54, CALCRL, CASKIN2, CDH5, CLDN5, COL4A3, CTTNBP2NL, DRAM1, EGFL7, EHD4, ELTD1, ENG, ENTPD1, EPAS1, EPHB4, ERG, ESAM, FAM110D, FGD5, GPR116, HBEGF, HILPDA, HSPA12B, ICAM1, ICAM2, KDR, KIFC1, LATS2, LRRK1, MMRN2, MYO1B, NOTCH4, NPNT, NPR3, PALD1, PECAM1, PLTP, PTPRB
Inflammation	IFNG, IFNGR1, IFNGR2, IL10, IL12A, IL12B, IL12RB1, IL12RB2, IL13, IL17A, IL17F, IL18, IL18R1, IL18RAP, IL1A, IL1B, IL2, IL21, IL21R, IL22, IL23A, IL23R, IL2RG, IL4, IL4R, IL5, IL6, JUN, NFKB1, RELA, RORA, RORC, S100A8, S100A9, STAT1, STAT3, STAT4, STAT6, TGFB1, TGFB2, TGFB3, TNF
